# 

**Published:** 1894-05-12

**Authors:** 


**Notices of Births, Deaths, and Marriages** are inserted in this column at a charge of 2s. 6d. for an announcement not exceeding four lines.

**Notice to Advertisers and Subscribers.**

All Advertisements, Orders for Copies of The Hospital, and Business Communications should be addressed to **The Publisher, NOT TO THE EDITOR,** The Hospital, 428, Strand, W.C.

The Cost of Subscription, post free, is as follows:—Per week, 2½d.; per quarter, 2s. 8d.; per half-year, 5s. 3d.; per annum, 10s. 6d. Foreign:—Per Annum, 12s. 8d.; payable, in all cases, in advance.

NOTICE TO CORRESPONDENTS.

All MS., Letters, Books for Review, and other matters intended for the Editor should be addressed The Editor, The Lodge, Porchester Square, London, W.

The Editor cannot undertake to return rejected MS., even when accompanied by stamped directed envelope.

**"Burdett's Hospital Annual," 1894.**

Fifth year of publication. 900 pp. Boards, gilt lettering. A most complete guide to British, Colonial, Indian, and American Hospitals, Dispensaries, Nursing and Convalescent Institutions, and Asylums. Price 5s. Published by The Scientific Press (Limited), 428, Strand, W.C.

**NOTICE.—The Index for Vol. XIV. (ending Sept. 30th, 1893) is on sale at the Office of this Journal, price 2½d., post free.**

### MEDICAL VACANCIES AND APPOINTMENTS.

### VACANCIES.

The following vacancies are announced this week, the amount of salary in each case being given after the name of the institution:

### Resident Medical Officers.

Chesterfield and North Derbyshire Hospital and Dispensary, Chesterfield.—Resident Junior House Surgeon and Dispenser. Salary £50 per annum, with board apartments, and laundress. Applicatiens to the Secretary by May 17th.

Durham County Hospital.—House Surgeon. Salary £100 per annum, with board and lodging. Appointment for two years. Applications to V. K. Cooper, Honorary Secretary, 16, South Bailey, Durham, by June 1st.

Flintshire Dispensary.—House Surgeon. Salary £120 per annum, with furnished house, rent and taxes free; also coal, light, water, and cleaning, or, in lieu thereof, £20 per annum. Knowledge of Welsh desirable. Applications to W. T. Cole, Secretary, Board Room, Bagillt Street, Holywell, by May 15th.

Fisherton Asylum.—Asssistant Medical Officer, not more than 30 years of age and single. Salary £100 per annum, with board, lodging, and washing. Applications to Dr. Finch, Salisbury.

Hospital for Sick Children, Great Ormond Street, Bloomsbury, W.C.—House Physician and House Surgeon, unmarried. Appointments for six months. Salary, in each case, £20, with board and residence in the hospital. Applications to the Secretary by May 25th.

Kent County Asylum, Chartham, near Canterbury.—Junior Assistant Medical officer, unmarried. Salary £125 per annum, with furnished apartments, board, and attendance. Applications to Allen Fielding, Clerk to the Committee of Visitors, Solicitor, by May 14th.

Manchester Hospital for Consumption and Diseases of the Throat and Chest, Bowdon, Cheshire.—Resident Medical Officer. Salary £60 per annum, with board, apartments, and washing. Applications to C. W. Hunt, Secretary, by May 15th.

Nottingham Borough Asylum.—Second Assistant Medical Officer, unmarried. Salary £100 per annum, with board, apartments, and washing. Applications to the Medical Superintendent by May 21st.

Vestry of St. Margaret and St. John, Westminster.—Medical Officer; not less than 25, or more than 45, years of age. Salary £250 per annum. Applications, marked on the envelope "Medical Officer," to be delivered at the Town Hall, Westminster, S.W., by May 21st.

York Dispensary.—Resident Medical Officer, unmarried. Salary £150 per annum, with furnished apartments, coal, and gas. Applications to Mr. W. Draper, De Grey House, York, by May 15th.

### Non-Resident Medical Officers.

Courcies Dipensary District, Ballinspittle, Ireland,—Medical Officer. Salary £100 per annum as Medical Officer, and £15 per annum as Medical Officer of Health. Applications to Mr. Patrick O'Connell, Honorary Secretary, Old Head, Ballinspittle, by May 15th.

Dental Hospital of London and London School of Dental Surgery, Leicester Square.—Demonstrator. Honorarium £50 per annum. Applications to Morton Smale, Dean, by May 14th.

Dental Hospital of London, Leicester Square.—Two Assistant Anæsthetists. Applications to J. Francis Pink, Secretary, by May 14th.

Derby Asylum, Mickleover.—*Locum Tenens* for two months and a-half, qualified and registered. Terms £2 2s. weekly, with board, &c., and travelling expenses to asylum. Applications to the Medical Superintendent.

General Infirmary, Leeds.—Pathological Curator. Honorarium 20 guineas per annum. Applications to Mr. Littlewood, Secretary, by May 14th.

Gordon Hospital for Fistula, 276, Vauxhall Bridge Road, S.W.—Two Honorary Surgeons, must be F.R.C.S.Eng. Applications to Mr. St. Leger Bunnett, Secretary, by May 21st.

Newton Abbot Union.—Medical Officer for the Workhouse. Salary £50 annum. Applications to John Alsop, Clerk, Union Offices, East Street, Newton Abbot, by May 15th.

Westminster Hospital, Broad Sanctuary, S.W.—Surgical Registrar. Must be F. or M.R.C.S.Eng. Appointment for twelve months. Salary £40 per annum. Applications to Sidney M. Quennell, Secretary, by May 22nd.

### The Hospital Advertiser.

London: THE SCIENTIFIC PRESS (Limited). 428, Strand, W.C.

London: The SCIENTIFIC PRESS (LIMITED), 428, Strand, W.C.

### THE SCIENTIFIC PRESS LIST.

Second Edition. Nearly Ready. Illustrated, Crown 8vo., 400 pp., price 5s. [*In the Press.*

### HELPS IN SICKNESS AND TO HEALTH.

By HENRY C. BURDETT,
Author of "Hospitals and Asylums of the World," &c. An invaluable work for every household.

"It would be difficult to find one which should be more welcome in a household than this unpretending but most useful book."—*The Times.*

"We can heartily recommend this little epitome of useful information to all who desire to have at hand, in the most accessible form, a ready guide to tell them where to go and what to do, without a moment's doubt or loss of time, where time is so valuable that a few minutes or an hour lost may be irrecoverable in the mischief resulting."—*Spectator.*

"We have often desired to obtain, either for ourselves or friends, the very information this book supplies. We therefore can, with justice, commend this synopsis to the profession and to the public; indeed, we feel that no medical or general library can be complete without such a book of ready reference."—*Lancet.*

"This books fill a gap in popular sanitary literature by providing within the compass of one volume of very moderate size a useful collection of facts not easily found elsewhere, unless a sanitary library be at hand."—*British Medical Journal.*

"One great merit of this manual is that its advice rests on the sound basis of common-sense. The work takes a wide range, but it is thoroughly practical throughout."—*Daily Chronicle.*

"It would be difficult to speak too highly in praise of this little work, which is a marvellous compendium of useful knowledge for a very wide variety of readers. Of its kind we regard it with a sense of almost unqualified admiration."—*Medical Press.*

"It is throughout replete with common-sense, full of valuable information, though free from technical details, giving just so much as everyone ought to know and no more, and is, too, written in an easy and agreeable style."—*Medical Times.*

IMPORTANT NEW HANDBOOK OF MIDWIFERY.
18mo., profusely illustrated with original cuts, tables, &c.; about 260 pp., handsomely bound in leather. Price 6s.,

### A PRACTICAL HANDBOOK OF MIDWIFERY.

By FRANCIS W. N. HAULTAIN, M.D.

This Handbook is produced in a portable and convenient form for reference, and it is intended mainly as a practical help to the busy practitioner. It is bound in roan, of a size convenient for the pocket.

To Students it brings forward succinctly the chief points of practical importance in the study of Obstetrics.

"No practical handbook published recently will, we believe, be more welcome to students than this on Midwifery just issued. Within a very attractive leather cover are some 240 pages, containing a veritable store of receipts and hints well up to date. The author is coming to be recognised as a successful teacher in the Edinburgh Medical School, and this volume is one which will add to his reputation. Special mention may be made of the chapters dealing with Extra-uterine, Gestation, Contracted Pelves, and Puerperal Dangers. It will, we anticipate, prove a help in the many difficulties students and practitioners have to face in Obstetrics."—*The Student.*

"This little work is intended to be a practical help to the busy practitioner, and will be found useful for refreshing the memory. Though written in tabular form, it contains a large amount of information, and the rules for treatment are succinctly expressed. It should prove of great use to the junior practitioner, who might with advantage take it in his pocket to the earlier cases, and to the lecturer as a complete synopsis. For example, the whole subject of pelvic presentations is given as completely as in many larger works. Some good diagrams elucidate the text. We can heartily recommend this book as a practical help."—*Glasgow Medical Journal.*

"MR. BURDETT'S MONUMENTAL WORK."—*The Times.*

In FOUR VOLUMES: Crown quarto, handsome cloth gilt, bevelled edges, PROFUSELY ILLUSTRATED with PLANS and DESIGNS. Together with a portfolio containing SOME HUNDREDS of PLANS. Price £8 8s. complete:—

### HOSPITALS AND ASYLUMS OF THE WORLD:

Their Origin, History, Construction, Administration, Management, and Legislation; with Plans of the Chief Hospitals, Asylums, Convalescent Institutions, Special and Infectious Hospitals, Nurses' Homes, and Medical School Buildings, in addition to those of all the Hospitals of London in the Jubilee Year of Queen Victoria's Reign, accurately drawn to a Uniform Scale.

By HENRY C. BURDETT.

Vols. I. and II.—ASYLUMS—HISTORY and ADMINISTRATION, CONSTRUCTION, PLANS, and BIBLIOGRAPHY. Price £4 10s.

Vols. III. and IV.—HOSPITALS—HISTORY and ADMINISTRATION, CONSTRUCTION, PLANS, and BIBLIOGRAPHY. With Portfolio containing some Hundreds of Plans. Price £6.

THE ENTIRE WORK COMPLETE, Price £8 8s.

Of the small edition printed a limited number of copies still remain, and Institutions, Libraries, and Clubs have therefore an opportunity to add this remarkable work to their libraries which cannot much longer continue.

A limited number of Portfolios of Plans will be supplied separately at £3 3s. each.

"The completed publications of this important works marks an era in the history of hospital literature. To students of hospital management and economy this book will be a necessity; architects emulous of hospital construction will find it simply invaluable: whilst the lay reader . . . will find ample reward for his courage. Mr. Burdett has had more than a quarter of a century's practical experience of the administration of hospitals and kindred institutions. Both he and his readers are to be congratulated on the happy attainment of his task."—*The Spectator.*

Demy 8vo., nearly 200 pp., cloth gilt, profusely illustrated with over 70 special cuts, price 6s., post free.

### ART OF MASSAGE. By A. Creighton Hale.

A complete text-book of this valuable art. The most practical, simplest, and generally useful work published on this subject. Suitable for self-tuition.

"A volume on the 'Art of Massage,' from the pen of one of its foremost advocates in this country. . . . Admirably illustrated."—*Westminster Review.* "The latest and most complete book we know on Massage."—*Queen.* "A clear exposition of the science. A book to be read by all interested."—*Publishers' Circular.*

Third Edition, about 400 pp., profusely illustrated, crown 8vo. [*In the Press.*

### COTTAGE HOSPITALS, General, Fever, and Convalescent: Their Progress, Management, and Work. By Henry C. Burdett.

Author of "Hospitals and Asylums of the World," "Pay Hospitals of the World," "Hospitals and the State," "Cottage Hospitals: General, Fever, and Convalescent, with Fifty Beds and Under," "The Relative Mortality of Large and Small Hospitals," "Helps to Health," "Burdett's Hospital Annual and Year Book of Philanthropy," and Editor of The Hospital.

THE FEEDING OF INVALIDS.
Now Ready. Demy 8vo., cloth gilt, 260 pp. Price 3s. 6d.,

### ART OF FEEDING THE INVALID.

By a Medical Practitioner and a Lady Professor of Cookery.

A book that the practitioner can place in the hands of the nurse as a guide in his absence.

Matrons of institutions, and all engaged in the care of the sick, have found it invaluable as a daily guide.

This important work fully and clearly describes the nature and tendency of diseases most frequently met with, and gives a general outline upon which the diet of the invalid should be regulated. Corresponding with the chapters are lists of new and original recipes, which are intended for daily use and reference.

The labour and anxiety of providing suitable diet for invalids will, by the use of this book, be reduced to a minimum.

Among the subjects treated are the folllowing: The Feeding of Invalids Generally; Food; Food in Acute Febrile Diseases, in Convalescence and Chronic Illness, in Indigestion, in Gout, in Constipation and Diarrhœa, in Liver and Kidney Diseases, in Diabetes, in Tuberculosis and Consumption, in Corpulence, and for Children.

"The Art of Feeding the Invalid" has the approval of Members of the Faculty, its Medical author being widely known as the author of a standard text-book.

The Recipes are entirely new, having been especially written by a Professional Cook who holds diplomas and has had vast experience as a lecturer.

Now Ready. Demy 8vo., about 150 pp., profusely illustrated with Coloured and other Plates and Drawings. Price 6s. net,

### LECTURES ON GENITO-URINARY DISEASES.

By J. C. OGILVIE WILL, M.D., C.M., F.R.S.E.,
Consulting Surgeon to the Aberdeen Royal Infirmary, and Examiner in Surgery in the University of Aberdeen.

Contents.—Urethral Fever and Catheter Fever—Treatment of Retention of Urine—Gleet and its Treatment—On Varicocele—On Hydrocele—The Treatment of Syphilis—Appendix—Prescriptions for Syphilis, &c., &c., &c.

